# Selenium – a scoping review for Nordic Nutrition Recommendations 2023

**DOI:** 10.29219/fnr.v67.10320

**Published:** 2023-12-28

**Authors:** Jan Alexander, Ann-Karin Olsen

**Affiliations:** Norwegian Institute of Public Health, Oslo, Norway

**Keywords:** selenium, selenoprotein, trace elements, antioxidants, nutrition recommendations

## Abstract

Selenium is an essential trace element in humans, critical to the normal physiology in all animal species. The main form of selenium in food is selenomethionine, selenocysteine and a variety of organic compounds, while inorganic salts mainly occur in food supplements. In animals and humans, selenium occurs as selenocysteine in selenoproteins encoded by 25 genes (specific selenium pool). Several selenoproteins are part of the antioxidant enzyme system and serve as oxido-reductases and in thyroid hormone regulation. SelenoproteinP (SELENOP) transports selenium to peripheral tissues, is the main plasma selenoprotein, and has been used as biomarker of selenium status and intake. SELENOP in plasma represents a saturable pool of selenium and is maximised at a selenium concentration in plasma of about 110 µg/L or an intake of selenomethionine at about 1.2 µg/kg body weight in adults. In Finland, with an estimated selenium intake of 88 µg/day in men and 68 µg/day in women, the average selenium concentration in plasma is about 110 µg/L. Imported wheat from selenium rich areas is an important dietary source in Norway. Dietary intakes in the Nordic and Baltic area vary from 39 to 88 µg/day in men and 22 to 68 µg/day in women, the highest levels were from Finland. Most intervention trials on the effect of selenium supplementation on health outcomes have been carried out in ‘selenium-replete’-populations and show no beneficial effect, which from a nutritional point of view would rather not be expected. Some intervention studies conducted in populations low in selenium have showed a beneficial effect. Observational studies suggest an inverse relationship between selenium status and risk of cardiovascular diseases (CVDs), cancer and all-cause mortality, and some other outcomes at low levels of intake (<55 µg/day) or in plasma or serum (<100 µg/L). However, a lack of quantitative data and inconsistencies between studies precludes these studies to be used to derive dietary reference values. At high intakes above 330 to 450 µg/day selenium may cause toxic effects affecting liver, peripheral nerves, skin, nails, and hair. An upper tolerable level (UL) of 255 µg selenium/day in adults was established by EFSA.

## Popular scientific summary

- Selenium is an essential trace element and critical to normal physiology.
- In the body, selenium is needed for synthesis and maintenance of selenoproteins, which i.a. have antioxidant properties and regulate thyroid homones.
- Levels of SelenoproteinP in plasma is the most appropriate biomarker of selenium status.
- The level of selenium in food varies with the selenium content of soil, which is poor in most areas of the Nordic and Baltic countries.
- Increased selenium intake may possibly reduce risk of cardiovascular disease and some cancers in populations with low selenium status, but not in selenium replete populations.

Selenium is an essential trace element in humans and is critical to the normal physiology in all animal species (1–4). A recommended intake (RI) was previously set for selenium in the Nordic Nutrition Recommendations 2012 (NNR2012) (5) and an adequate intake (AI) was set by European Food Safety Authority (EFSA) in 2014 (4).

Selenium has an atomic weight of 78.96 and belongs to group 16 in the periodic table placed below oxygen and sulphur (1). Elemental selenium has both metallic and non-metallic properties, and is insoluble in water. Selenium dioxide, formal oxidation state +4, forms selenous acid in water, whereas selenium trioxide, formal oxidation state +6, forms selenic acid in water, and their salts, selenite and selenate are the main forms in water and soils. Selenium forms stable bonds with carbon and in nature, plants, microorganisms, yeast, and other organisms form a large variety of low molecular weight organic selenium compounds. In most of these, selenium occurs as selenide, and they are usually water-soluble. There are great variations in selenium soil content around the world, from poor to very rich (1, 2). Although higher plants do not need selenium, they readily take up and reduce inorganic selenium salts from soil by different mechanisms and using pathways of sulphur analogues. Plants that tend to accumulate sulphur, for example mustards, cabbages, and other plants in the brassica family, also accumulate selenium. In addition to formation of numerous organic molecules, plants incorporate selenium into proteins mainly as selenomethionine, but also as selenocysteine at the expense of its sulphur analogues, methionine, and cysteine. As free selenocysteine is toxic to plants, it is either incorporated into proteins or detoxified by methylation and stored as methylselenocysteine. High levels of selenium in soil may be toxic to plants, but some plants appear to tolerate that. The main form of selenium in food is selenomethionine. In animals and humans, selenium occurs as selenocysteine in selenoproteins where it is incorporated by a specific biochemical mechanism. When selenium is consumed as selenomethionine, it can also be non-specifically incorporated as a substitute for methionine in proteins in all tissues.

All selenoproteins contain selenium as the amino acid selenocysteine, amino acid no. 21. In selenocysteine the thiol group in cysteine is substituted by a selenol group, which has a much lower pKa (5.2 vs. 8.3) and a higher reactivity than the corresponding thiol. It can therefore interact more rapidly with reactive oxygen species, which is why several selenoproteins are part of the antioxidant enzyme system and serves as oxido-reductases.

As selenium from soil is incorporated into crops for food and feed, food for humans will vary with soil selenium content accordingly, resulting in both selenium overt deficiency, subclinical deficiency, adequate intake and toxicity of populations living in different geographical areas.

The aim of this scoping review is to describe the totality of evidence for the role of selenium for health-related outcomes as a basis for setting and updating dietary reference values (DRVs) for the Nordic Nutrition Recommendations 2023 (NNR2023) (Box 1).

*Box 1.* Background papers for Nordic Nutrition Recommendations 2023

- This paper is one of many scoping reviews commissioned as part of the Nordic Nutrition Recommendations 2023 (NNR2023) project (7).
- The papers are included in the extended NNR2023 report but, for transparency, these scoping reviews are also published in Food & Nutrition Research.
- The scoping reviews have been peer reviewed by independent experts in the research field according to the standard procedures of the journal.
- The scoping reviews have also been subjected to public consultations (see report to be published by the NNR2023 project).
- The NNR2023 committee has served as the editorial board.
- While these papers are a main fundament, the NNR2023 committee has the sole responsibility for setting dietary reference values in the NNR2023 project.

## Methods

This scoping review builds on information given in the former NNR2012 (5) and in the assessment of selenium by EFSA in 2014 (4). The review follows the protocol developed within the NNR2023 project (6, 7).

Updating of systematic reviews on selenium published since NNR2012 was conducted by a general literature search in PubMed using the search string: “selenium[MeSH Terms] AND (“2011”[Date – Publication] : “3000”[Date – Publication]) AND Humans[Filter] AND (“Diet” OR “Dietary” OR “Food” OR “Nutrition” OR “Nutritional”) AND systematic review[Publication Type]”. Date of search was 23 July 2022. In all, 84 citations were retrieved from the search, and 10 publications were included in this review. None of these was used as direct evidence in the derivation of a DRV. No *de novo* systematic review was performed for the NNR2023 project (8).

## Physiology

The description of selenium physiology is largely based on reviews (1, 4, 9, 10).

### Chemical aspects, biochemistry

The main selenium species in food is selenomethionine, selenocysteine, and minor amounts of many different low molecular weight organic selenium compounds. Food contains less of selenium salts, selenite and selenate, but these are used in food supplements. Selenomethionine and selenocysteine in food is provided mainly bound in proteins.

### Absorption

About 70–80% of selenium from various food items is absorbed. Selenium from organic sources appears to be more bioavailable than that from inorganic sources (11). Experimental studies have shown poor bioavailability of selenium from some fish species. In humans, selenium has been shown to be readily available from Baltic herring and rainbow trout (12, 13). Other human studies have suggested reduced bioavailability of selenium from fish compared with other selenium-containing foods (14, 15). In addition to selenomethionine, fish also contains selenoneine, a compound that apparently does neither support selenoprotein synthesis nor increase plasma selenium, which could be a reason for the observed differences in ability to raise plasma selenium (1).

**Fig. 1:**
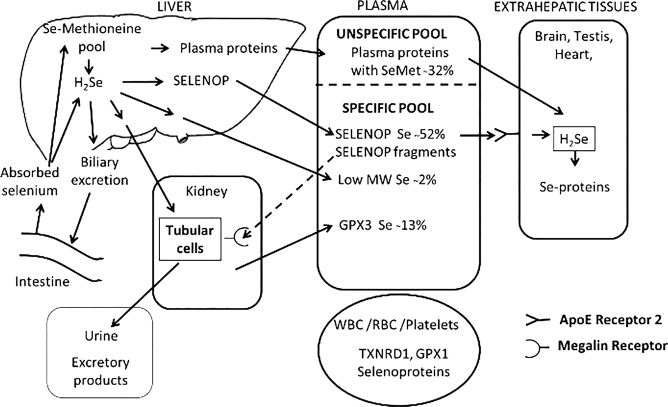
Overview of selenium absorption, distribution, and excretion. The distribution of selenium in plasma is representative for a selenium replete individual (9). Reproduced with permission (1).

The absorption of selenium compounds occurs mainly in the duodenum and most water-soluble selenium compounds are readily absorbed. The absorption is not influenced by dose and nutritional status. Selenomethionine, methylselenocysteine, and selenocysteine are actively transported using amino acid transporters, and other selenium compounds appear to use similar transport mechanisms as their sulphur analogues. While selenate is almost completely absorbed, selenite absorption seems to be slower and less efficient. For an overview of selenium absorption, distribution and excretion, see Fig. 1.

### Distribution and storage

Results from human studies suggest that selenium kinetics is like that of laboratory animals. Following absorption, water-soluble selenium compounds are transported via the portal vein to the liver before they reach systemic circulation and distribute to most organs. The degree of whole-body retention is inversely related to dietary level of selenium. Surplus of selenomethionine can be non-specifically incorporated in proteins in place of methionine and thereby enter a non-regulated storage pool of selenium (also denoted the methionine pool).

The distribution of selenium to extrahepatic tissues is regulated to support synthesis of selenoproteins. SelenoproteinP is an extracellular selenoprotein produced in the liver and secreted to plasma. It contains up to 10 selenocysteine residues and is the major circulating form of selenium, constituting about 50 to 60% of plasma selenium in replete individuals. In extrahepatic tissues, SELENOP is taken up by means of the low-density lipoprotein receptor-related protein-8/ apolipoprotein E receptor-2 (LPR-8/ApoER-2) and processed for selenoprotein synthesis. SELENOP fragments are also secreted from the liver, and after entering into primary urine, they are taken up by kidney tubular cells by the low-density lipoprotein receptor-related protein 2 (LRP-2)/megalin. Another selenoprotein, glutathione peroxidase 3 (GPX3), is found in the basement membrane surrounding the proximal tubuli. It is secreted to plasma from the kidney but occurs in far lower amounts in plasma (10–20% of total Se) than SELENOP. Both of these proteins occur in human breast milk and are important for supplying the breastfed child with selenium (16). Both selenoproteins are also important for transplacental transfer of selenium to the foetus. Selenium may also in a non-regulated fashion be distributed to other tissues and breast milk via plasma and milk proteins containing selenomethionine in addition to low molecular weight selenium compounds. The amount of selenium in breast milk declines over time during lactation.

### Metabolism

**Fig. 2:**
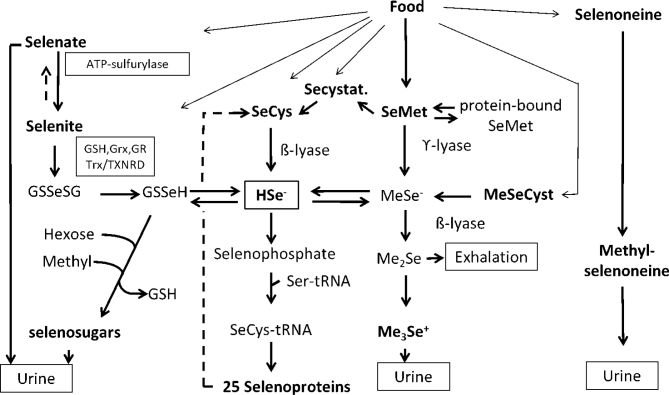
Overview of selenium biotransformation. Glutathione (GSH, glutathione reductase (GR) glutaredoxin (Grx), thioredoxin (Trx), thioredoxin reductase (TXNRD), selenocysteine (SeCys), methylselenocysteine (MeSeCys), selenomethionine (SeMet), selenocystathione (SeCystat), seryl-tRNA (Ser-tRNA), methylselenide (MeSe^-^), dimethylselenide (Me_2_Se), trimethylselenonium ion (Me_3_Se^+^). Reproduced with permission (1).

Hydrogen selenide (HSe^-^) is a central intermediate metabolite to which several biotransformation pathways converge (1, 9). Simple selenium salts selenate (Se^6+^) and selenite (Se^4+^) are reduced using glutathione (GSH) as essential cofactor via GSSeSG and GSSe^-^ catalysed by GSH reductase, thioredoxin (Trx), thioredoxin reductase (TXNDR), and glutaredoxin (Grx). In the presence of oxygen and at high levels, selenides may redox cycle at the expense of reducing equivalents (i.e. Nicotinamide adenine dinucleotide phosphate hydrogen [NADPH]) generating reactive oxygen species. The reduction of selenate to selenite is slow and is not fully characterised. Surplus of selenate circulates in plasma and is excreted unchanged in urine. For an overview of selenium biotransformation, see Fig. 2.

At high and toxic doses, hydrogen selenide (HSe^-^) increases, but is detoxified by sequential methylation to dimethylselenide and trimethylselenonium (TMSe^+^). At lower doses, surplus of HSe^-^ is via GSSeH conjugated to hexoses and methylated to excretable sugar-metabolites that is the dominating pathway. There is a polymorphism in the methylating gene, making a fraction of the population (6–20%) TMSe^+^ producers also at low doses of selenium.

Selenomethionine is converted to selenocysteine by cystathionine β-synthase and ϒ-lyase via selenocystathionine. Alternatively, it can be converted by intestinal bacteria or by β-lyase in cells to methylselenol. Selenocysteine is converted to HSe^-^ by selenocysteine β-lyase, which is an important mechanism in the recycling of selenium from selenoproteins. Selenomethionine cannot be formed in the body and therefore only selenium ingested as selenomethionine can enter the unspecific selenomethionine pool in proteins.

Selenoneine, an ergothioneine analogue that occurs in fish, does not seem to contribute to hydrogen selenide production, but is methylated and excreted.

### Selenoprotein synthesis

Selenocysteine cannot be directly incorporated into selenoproteins as selenocysteine in selenoproteins is synthesised *de novo* from selenide on its own selenocysteine-tRNA, which is initially charged with serine and subsequently converted to selenocysteine-tRNA by selenophosphate. Selenocysteine-tRNA is incorporated into selenoproteins by a specific machinery guided by UGA codon that is present in all selenoprotein mRNAs. Following recoding the UGA codon from a stop codon to fit selenocysteine-tRNA, it is incorporated by the selenocysteine insertion (SECIS) element at the 3’-untranslated region of the mRNA and recruited proteins, that is, SECIS-binding protein2 (SBP2).

In humans there are 25 genes coding for selenoproteins and isoforms (alternative splicing and posttranslational modifications), which all contain selenium as selenocysteine. Except for SELENOP, which contains 10 selenocysteine residues, all the selenoproteins contain one selenocysteine. Compared to thiol groups, selenol groups are ionised at physiological pH and are much more reactive. Based on studies in experimental animals, there seems to be a hierarchy of selenoproteins expressed, housekeeping- and stress-related selenoproteins, depending on nutritional status. The regulation of expression of different selenoproteins is influenced by various biochemical mechanisms, which have not been fully elucidated. Genetic polymorphisms may also modulate the expression and function of selenoproteins (17). This means that at suboptimal intake of selenium, expression of stress-related selenoproteins, for example, selenoproteins protecting against oxidative stress and supporting adequate immune response, might be compromised.

### Excretion

Under most conditions, urine is a major excretory pathway with the greatest excretion during the first day after ingestion. Urinary excretion constitutes about 40–60% of the intake, but it depends on the nutritional status and selenium compound provided. Faecal excretion consists of unabsorbed selenium and selenium from turnover of intestinal mucosal cell. Studies in rats show that selenium excreted in bile is mostly reabsorbed. Some authors have estimated faecal selenium to be up to 50% of that in urine. Generally, inorganic selenium salts are less well retained than organic selenium, selenomethionine.

The pattern of selenium compounds in urine is dependent on nutritional status and selenium species taken in. At moderate doses, selenosugar conjugates dominate in addition to minor amounts of other low molecular weight selenium species. The majority of selenate not reduced to selenite is excreted unchanged. In 6–20% of the population, TMSe^+^ is the dominating species in urine. Faecal selenium in humans has not been characterised.

At high and toxic doses of selenium with excess formation of HSe^-^, methylation with exhalation of volatile dimethyl selenide becomes important and may even predominate.

### Molecular functions, molecular mechanisms

The physiological functions of selenium are mediated by its presence in selenoproteins. Selenoproteins have a variety of functions that all together are vital for normal development and physiology in all higher animals including humans. Many of the selenoproteins have been ascribed a biological function, but for several their functions have not yet been fully characterised (1).

Selenoproteins that are antioxidative oxidoreductases comprise five glutathione peroxidases (GPXs) that converts hydroperoxides and organic peroxides (e.g. lipoperoxides) to water or alcohols: GPX1, cytosolic presence and widely expressed; GPX2, gastrointestinal tract; GPX3 plasma and extracellular fluids, renal proximal tubuli where it is produced; GPX4, acts on phospholipoperoxides and protects cellular membranes, widely expressed, particularly in the heart muscle and function as a structural protein in sperm; GPX6, reduced peroxides in olfactory tissue and embryo. Three thioredoxin reductases (TXNRDs) control redox status of thioredoxins and glutaredoxins that play key roles in cellular redox regulation: TXNRD1, cytoplasmic and nuclear locations, widely distributed, participate in redox regulation, support ribonucleotide reductase and DNA synthesis; TXNDR2, mitochondria, widely distributed, redox regulation; TXNDR3, redox regulation, testis.

Three iodothyronine deiodinases (DIO1, DIO2, DIO3) with different tissue distribution control central and peripheral activation and inactivation of thyroid hormones. DIO2 converts thyroxin to 3-iodo thyronine in peripheral tissues and organs, and in the central nervous system it plays an important role in the feedback regulation of thyroid hormone production.

SelenoproteinP is mainly synthesised in the liver (about 75%) and secreted to plasma. Different from other selenoproteins that have 1 selenocysteine, it has 10 selenocysteine residues. It has a dual role; it transports selenium to peripheral tissue, and has antioxidative properties and appears to play a role in protecting circulating lipoproteins against oxidation to more toxic species. Methionine-R-sulfoxide reductase B1 (MSRB1), reduces oxidised methionine, and plays a role in macrophages and innate immune functions. Selenophosphate synthetase 2 (SEPHS2) is involved in synthesis of selenocysteine-tRNA.

In addition to DIO2 and MSRB1, selenoprotein F (SELENOF), selenoprotein M (SELENOM), selenoprotein N (SELENON), selenoprotein S (SELENOS), and selenoprotein T (SELENOT), all reside in the endoplasmic reticulum. Their functions are not fully elucidated. They seem to be involved in redox control, endoplasmic stress, control of calcium flux (SELENOK, SELENON), and control of misfolded proteins (SELENOS, SELENOK). SELENOK is important for proliferation of immune cells in cancer protection apparently via its impact on calcium flux (4, 18).

SELENON, selenoprotein O (SELENOO) residing in mitochondria, and selenoprotein W (SELENOW) in cytosol are important for skeletal and cardiac muscle growth and function, brain, and bone mass. Selenoprotein H (SELENOH) resides in the nucleus, where it protects DNA synthesis, and in mitochondria.

Transgenic and knockout mouse models have been used to elucidate functions and impact on pathology of selenoproteins. For example, GPX4 knock-out mice are infertile as they miss a key structural sperm protein. In mice, complete deletion of selenocysteine tRNA is embryonic lethal.

In humans, various mutations and polymorphisms affecting genes coding for selenoproteins or their synthesis provide evidence for their physiological role. For example, mutations in SBP2, which play a key role in the synthesis of all selenoproteins, have been shown to be associated with abnormal thyroid hormone metabolism, delayed bone maturation, congenital myopathy, impaired mental and motor coordination development, and reduced growth rate. For polymorphisms and mutations in genes coding for selenoproteins the phenotype will vary accordingly.

## Assessment of nutrient status

Several biomarkers of nutritional intake or status of selenium have been used. These include: selenium concentrations in body fluids: whole blood, plasma/serum, and urine; blood cells: red blood cells and platelets; and hair and nails. The enzyme activity or concentrations of selenoproteins in blood plasma/serum or in blood cells such as plasma SELENOP and plasma GPX3 or platelet GPX have also been used.

### Plasma/Serum and whole blood selenium

Selenium concentrations in plasma and serum are considered similar and comprised of selenoproteins and selenomethionine in plasma proteins. Whole blood selenium adds selenoproteins and selenomethionine in other proteins of leukocytes, platelets, and erythrocytes. Low molecular weight selenium compounds constitute usually only a minor part in plasma and whole blood (Fig. 1).

Dietary selenium intake is the major determinant of selenium plasma concentration. Other factors such as age, sex, smoking, and inflammation may also, but to a much lower extent, influence plasma selenium concentration.

While the expression and secretion of selenoproteins in plasma is regulated, there is no apparent regulation of selenium plasma concentration. The relationship between selenium intake and plasma selenium depends on the chemical composition of selenium compounds. Selenium species, inorganic salts, selenate and selenite, and other species that support selenoprotein synthesis will increase SELENOP and GPX3 and plasma selenium concentration until these have reached maximal expression and then level off. In supplementation studies in New Zealand women and Finnish men, participants received 200 µg selenium daily as selenate. Both in men and women plasma concentration increased from a mean value of 70 and 53 µg/L, respectively, and plateaued at about 110 µg/L (19, 20). In studies supplementing with selenite, plasma selenium plateaued around the same value, between 103 and 125 µg/L in women and men receiving 100 to 200 µg/day of selenite, respectively (21–23).

**Fig. 3:**
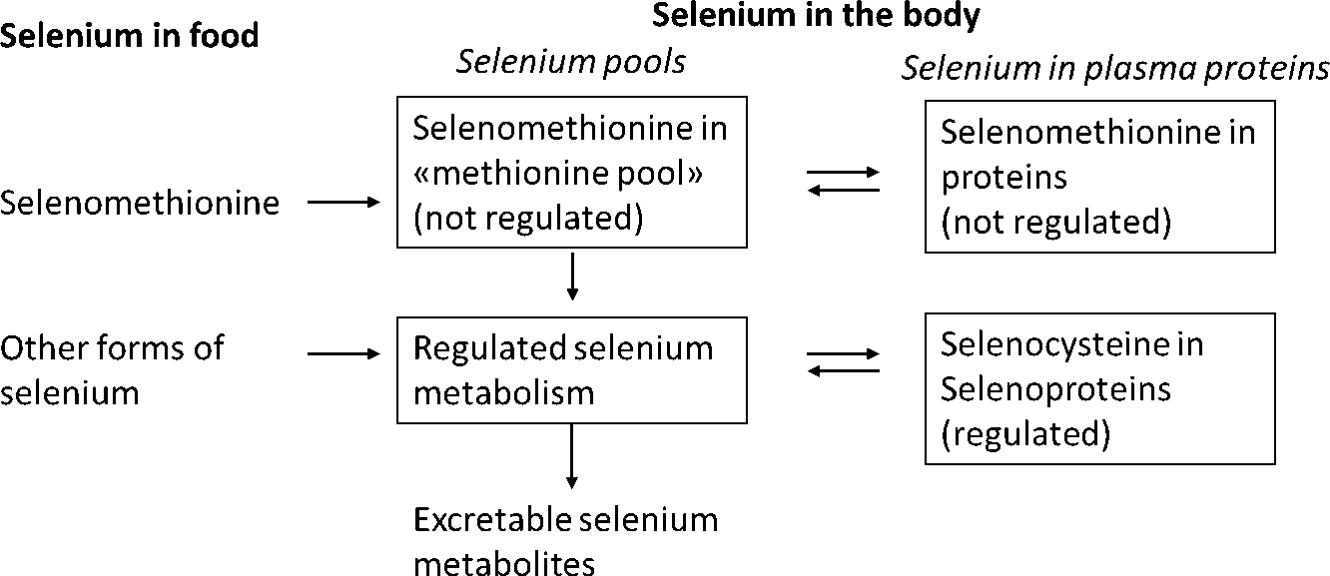
Illustration of regulated and saturable pool of selenium and relation to unregulated and non-saturable pool of selenium.

As selenomethionine is the main dietary species, a dietary intake of selenium or supplemental intake of selenomethionine or selenium yeast above the level required for maximal selenoprotein expression will further increase selenium plasma concentration until it at any constant intake reaches steady state after about 3 months depending on baseline level and dose (24, 25). For illustration, see Fig. 3.

The relationship between dietary selenium intake and plasma or whole blood selenium shows inter-individual variation and is also dependent on the nature of selenium compounds consumed. Hence, there is no established relationship between plasma or whole blood selenium and dietary intake. Hence, plasma selenium can only provide rough estimates of dietary selenium intake in a population.

### GPX activity in blood

Measurements of GPX 3 activity in plasma or GPX1 activity in corpuscular blood components that is, platelets, or GPX activity in whole blood are common biomarkers of selenium status. In studies from Finland (19, 23), it was estimated that platelet GPX activity reached a maximum at 100–115 µg/L. Other studies have shown that saturation of GPX3 activity has been reached from 70 to 90 µg/L of selenium in plasma, and do not further increase in individuals at concentrations around 100 µg/L (24, 26–29). GPX1 in platelets seems to plateau at a higher selenium concentration in plasma, 80 to 120 µg/L. In a study of the participants from the United Kingdom (UK) with a mean baseline selenium concentration of 95.7 ± 11.5 µg/L (29), there was no significant increase in platelet GPX upon supplementation, while there was an increase in SELENOP. Hence, there are limitations in the use of GPX activity as biomarker for selenium status. GPX activity seems to plateau at a lower selenium plasma concentration than that needed for plateauing of SELENOP concentration in plasma. Further, GPX3 originate from renal tubular cells and may rather be a measure of renal selenium status than the whole body status (30, 31).

### Plasma selenop concentration

Plasma SELENOP plays a key role in selenium transport to extrahepatic tissues and is mainly synthesised in the liver that control whole-body selenium homeostasis (9) (Fig. 1). The SELENOP supplementation studies have been conducted in populations with very variable selenium intake and status (30). Upon supplementation increasing doses of selenium, the concentration of SELENOP in plasma increases until it levels off; even if the selenium concentration in plasma still may increase. Hence, SELENOP in plasma seems to represent a saturable and regulated pool of selenium and an indicator of both intake and status up to a level of apparent saturation (24). The time required for supplementation up to SELENOP saturation varies with baseline selenium status, dose used and selenium species, and has in studies been reached between 4 weeks to 30–40 weeks (24, 29). In selenium replete populations, additional selenium intake will not lead to an increase in SELENOP (28, 32). For example, in a replete Unites States of America (US) population with a mean baseline plasma selenium concentration of 122 µg/L, supplementation with 200–600 µg/day increased plasma concentration, but did not further increase in SELENOP concentration (28). In a UK supplementation study lasting for 12 weeks and using doses of selenized yeast between 50 and 200 µg selenium/day on top of a habitual dietary intake of 55 µg/day, it was found that maximal expression of SELENOP was associated with a plasma concentration of 125 µg/L (29). As mentioned above, supplementation at mean baseline selenium concentration of 95.7 ± 11.5 µg/L in plasma still caused an increase in SELENOP. In a Chinese supplementation study in a selenium deficient population with an estimated habitual dietary intake of about 10 µg/day, supplementation with selenomethionine or selenite in daily doses up to 61 µg selenium for 20 weeks did not result in full expression of SELENOP in plasma (33). In their second study, lasting for 40 weeks, different doses of selenomethionine were administered (24). In the group given 35 µg selenium per day as selenomethionine on top of a habitual dietary intake of 14 µg/day (corresponding to 55 µg/day or 0.85 µg/kg bw/day), a plasma concentration of SELENOP of 4.9 mg/L was reached at a plasma concentration of 98 µg/L. Although not significantly different, the SELENOP concentration was still in the lower range of mean SELENOP concentrations (5.1–5.5 mg/L) reached at 40 weeks by total intakes of 69 and 93 µg selenium per day (corresponding to 1.19 and 1.44 µg/kg bw and day) and at selenium plasma concentration of 112 and 128 µg/L. Hurst et al. found that plasma concentrations of selenium required to achieve maximum SELENOP concentration in different studies varied between >90 to <130 µg/L (17). The composition of selenium species in the baseline diet and in the supplement given will likely have an impact on plasma selenium, as surplus of selenomethionine not needed for selenoprotein synthesis or bio-transformed to excretable derivatives may be non-specifically incorporated into plasma proteins and further raise selenium plasma concentration.

Determination of SELENOP has usually been done by means of immunoassays. As SELENOP occurs as different isoforms and different antibodies have been used, SELENOP concentrations from various studies cannot be directly compared. Ballihaut et al. (34, 35) described the technical difficulties of measuring SELENOP. In a replete US population SELENOP concentration was 5.5 mg/L, while in a deplete Chinese population from a low selenium area, and using the same assay, it was around 2.0 mg/L. Upon supplementation, it increased to around 5.5 mg/L (24, 28). In yet another study from the UK using the same assay, the supplemented groups reached up to a mean value of 6.7 mg/L. The interindividual variation is quite large as in the groups reaching SELENOP saturation, the concentration varied between 4.5 to 8.3 mg/L (29).

### Nail and hair selenium concentration

Selenium seems to accumulate in nail and hair along with sulphur, and has been used as a measure of long-term selenium intake or selenium status in observational studies. The concentration in nail and hair correlates with whole blood selenium, is associated with intake, and is regarded as a useful biomarker of long-term intake in epidemiological studies (4).

### Urinary selenium

Urinary excretion of selenium is highly variable, as surplus of selenium not going into selenoprotein synthesis or into proteins as selenomethionine is excreted into urine. Urinary excretion will also be influenced by the chemical nature of the selenium compounds taken in. At a constant dietary intake, selenium in plasma and urinary excretion appear to be closely related (28). Strong non-linear relationships with recent dietary intakes have been noted, indicating that urinary excretion may be an indicator of recent intake. However, considerable variation in urinary excretion limits its usefulness.

In summary, SELENOP in plasma is the most appropriate biomarker of the selenium status of the regulated selenium pool. Selenium in plasma is an indicator of dietary selenium intake. Maximal expression of SELENOP occurs at a plasma concentration of selenium at about 110 µg/L. Plasma and serum selenium are equivalent.

## Dietary intake in Nordic and Baltic countries

### Dietary sources

Geology and soil content of selenium, including pH and redox condition that determines its bioavailability, is of central importance for selenium in the food chain, the contents in crops and livestock, and resulting dietary intake. Soil selenium is poor in most areas of the Nordic and Baltic countries (36). Cereals and other crops grown in these areas, except for Finland after 1984, have low selenium content. In Finland, selenate has been added to agricultural fertilisers since 1984 (37). Inorganic selenium is converted to selenomethionine and minor amounts of numerous other organic species in plants for food and feed (1, 38).

In foods of animal origin, selenium occurs as selenocysteine and selenomethionine in proteins. Fodder low in selenium is generally enriched with selenite that only supports selenoprotein synthesis and has a limited effect on the selenium concentration of meat and dairy products. In muscle meat, 50–60% of the total selenium content might be in the form of selenomethionine, and about 20–30% of the selenium in beef and up to 50% in poultry might be selenocysteine (38). Fish contains both selenomethionine and selenoneine. Dairy products contain selenium mainly as selenocysteine and selenite. Supplementation could result in a wider spectrum of selenium species.

The inorganic forms selenite and selenate are used in dietary supplements and are not normally found in food.

### Dietary intake

Adding selenate to fertilisers has increased both human and animal intake of organic selenium in Finland (Table 1). The most important sources of selenium in the diet of Finns today are meat (which provides 40% of the selenium consumed), dairy products and eggs (25%), and cereal products (20%) (37).

**Table 1:** Dietary intake of selenium in adults 18 to 70/80 years.

	Selenium, μg/day		Selenium, μg/10 MJ	
	Men	Women	Men	Women
Denmark	61	46	55	55
Finland	88	68	96	95
Iceland	83	60	83.4	81
Norway^1^			63	
Sweden	50	42	56	59
Estonia	64.6	46.8	74	72
Latvia				
Lithuania	38.7	21.7	42	33

Source: Data in the table is taken from Lemming and Pitsi (44), where further details on intakes in various age groups for some countries can be found in supplementary data.

1 Mean for men and women, data from NNR2012 (5).

In Norway and Iceland, the intake of selenium has been influenced by high-selenium wheat imported from North America. In Norway, an increased use of domestically grown wheat during the last 20 years has reduced the average selenium intake of the population, as measured by a reduction in blood selenium concentrations (39).

In Sweden, selenium has been added to animal feed since the late 1980s, thereby increasing the intake from meat and dairy products. Fish and seafood are also important dietary sources (40). However, in fish selenium may also occur as selenoneine, which does not seem to support selenoprotein synthesis (1).

People from the Nordic area who regularly consume organically grown products might have a lower selenium intake, because the selenium concentrations in meat and milk from animals fed local organically grown feeds might have a lower content of selenium than meat and milk from animals conventionally fed. This also applies to vegetarians and vegans because plant foods might contain low levels of selenium. In Denmark, the mean selenium intake of vegans was compared with that of the general population. The intakes of vegan men and women were 33 and 25 μg Se/day, respectively, while men and women of the general population had intakes of 52 and 39 μg Se/day, respectively (41). Also, in an earlier study from Sweden, the intake of selenium among vegans was lower than that of omnivores (42). A similar difference was seen in Finnish vegans and non-vegetarians, 79 ± 65 μg Se/day and 149 ± 108 μg Se/day (mean ± standard deviation [SD]), respectively (43), but their intakes were generally higher than that in Denmark and Sweden. Differences in intake between vegans and non-vegetarians indicate that food of animal origin such as meat, eggs, and dairy products are significant sources of selenium in the Nordic countries.

### Plasma/Serum concentration of selenium

In Finland, the mean plasma concentration in adults was 110 µg/L (1.4 µmol/L) in 2010 (*n* = 60) (37), and in a small study the serum concentrations of selenium in non-vegetarians were 118, 105, 119 µg/L (1.5, 1.33, 1.51 µmol/L) in the median, 25^th^ percentile, and 75^th^ percentile (*n* = 18), respectively, and in vegans, 76.6 and 108.2 µg/L (0.97, 1.37 µmol/L) in the 25^th^ and 75^th^ percentile (*n* = 21), respectively (43). The lower values in vegans accord with the fact that about 70% of selenium intake in Finland comes from food of animal origin.

In recent Norwegian studies, median plasma/serum selenium concentrations varied between 64 to 95 µg/L, with 25-percentiles down to 51 µg/L (45–48). In an earlier study from a clinical chemistry laboratory, serum selenium concentrations were followed from 1995 to 2006. In that period, the mean concentration dropped from 1.35 to 0.95 µmol/L. The reduction was because of less import of selenium rich wheat (39).

In women (*n* = 548), in a Swedish birth cohort with a mean age of 30 years, the plasma selenium (median and 5–95^th^ percentile) was 67 (46–94) µg/L (49). The concentration was similar in group of elderly (*n* = 449), mean serum selenium: 67.1 µg/L (50).

In the Danish Prevention of Cancer by Intervention with Selenium (DK-PRECISE) from 1998 to 1999, participants, men and women aged 60–74 years (*n* = 491), had a baseline plasma selenium (mean and SD) of 86.5 (16.3) µg/L (51). During a 5-year intervention period, plasma values did not change in the placebo group. In a more recent study from 2010, similar levels were observed (52). In Greenland, the mean plasma concentration of selenium was 75.2 µg/L (53).

In Latvia, plasma selenium mean values between 80 and 90 µg/L, with ranges from 30 to 140 µg/L, have been reported (54, 55).

Plasma/serum selenium values from Estonia and Lithuania were not available.

## Health outcomes relevant for Nordic and Baltic countries

### Deficiencies

In animals, there are several well-defined syndromes related to selenium deficiency. This is less clear in humans as deficiency syndromes are often seen in combination with other contributing factors.

#### Keshan disease

Keshan disease is an endemic cardiomyopathy mainly occurring in children and young women of childbearing age (56). It was first described in 1935 in the Keshan region in China, where selenium levels in food are extremely low because of low soil selenium. The intake is usually below 20 µg per day. The incidence was dramatically reduced upon supplementation with selenite (57). A cardiotoxic coxsackievirus has also been implicated in the aetiology of Keshan disease (58). While Keshan disease is not directly relevant for the Nordic and Baltic countries, myopathies may be relevant, as cases of both cardiomyopathy and myopathy because of selenium deficiency have been reported both following long-term total parental nutrition without selenium supplementation and bariatric surgery (59–62).

#### Kaschin-Beck’s disease

Kaschin-Beck’s disease first described in 1849 is a chronic degenerative osteoarthritis affecting the cartilage of joints and growth plates, causing multiple symmetrical painful and inflamed joints associated with deformities. It is endemic in the severely selenium-deficient belt in China (63). Although selenium appears to have some preventive effect, the aetiology, pathogenesis and contributing factors are still unclear (64–66). This disease is not relevant for the Nordic and Baltic countries.

#### Cretinism because of joint selenium and iodine deficiency

Endemic myxoedematous cretinism has been described in schoolchildren living in the goitre area of central Africa because of joint low iodine and selenium intake (67). Whereas cretinism is not relevant; less is known about the impact of suboptimal intake of selenium and iodine on thyroid function in the Nordic and Baltic countries.

### Toxicities and adverse effects of selenium

Cases of acute toxicity following ingestion of selenium compounds, often supplements, have been reported in the literature (1). The symptoms include: nausea, vomiting, and garlic-like breath odour, hypotension, abnormal ECG, and neurological symptoms like tremor, muscle spasms, confusion, and coma. Chronic toxicity and clinical selenosis have been observed in areas with high levels of selenium in the soil. Typical symptoms are abnormal hair and nails including loss, mottled teeth, skin changes, affected peripheral nerves and liver toxicity. In Chinese studies, the lowest dose associated with chronic clinical selenosis was about 1,200 µg/day. While no signs of clinical selenosis could be observed at an intake of approximately 850 µg/day, increased prothrombin time – impaired liver protein synthesis – was observed above this level (1).

In a recent randomised controlled supplementation study from Denmark (51), an increased all-cause mortality after a follow-up of 15 years was observed among participants supplemented with 300 µg/day for 5 years above the dietary habitual intake of around 47 µg/day (68), while this was not seen in the group receiving 200 µg/day.

In the Selenium and Vitamin E Cancer Prevention Trial (SELECT), a randomised controlled study (RTC) from the US, it was noted that supplemented individuals with a mean intake of 330 µg/day (200 µg/day as supplement and a habitual dietary intake of 130 μg/day) had an increased risk of developing self-reported alopecia and dermatitis in comparison with individuals consuming dietary selenium only (69).

Possible adverse effects of supplementary selenium on type-2 diabetes are discussed below.

The EU Scientific Committee on Food in 2000 established an upper tolerable level (UL) for dietary selenium of 300 µg/day (70). EFSA recently updated the UL for selenium and a value of 255 µg/day in adults was established based on a lowest adverse effects level (LOAEL) of 330 µg/day in the SELECT study and a uncertainty factor of 1.3 (71).

### Nutrient related chronic diseases in Nordic and Baltic countries

#### Cardiovascular diseases

In two Finnish observational studies from the 1970s, low serum selenium levels (<45 µg/L) were associated with an increased risk of cardiovascular death (72). In an observational follow-up study from Denmark, it was found that men with serum selenium concentrations below 80 µg/L had an increased risk of ischaemic heart disease (73). Since then, several observational studies have been published, but showing conflicting results.

A Cochrane meta-analysis from 2013 included 12 supplementation trials lasting for more than 3 months with a total of 19,715 randomised participants (74). There were no statistically significant effects of selenium supplementation on all-cause mortality, CVD mortality, non-fatal CVD events, or all CVD events (fatal and non-fatal). Of note, most of the participants were from two studies from US, the Nutritional Prevention of Cancer Trial (NPC) (75) and the SELECT (69). In both studies, baseline selenium was high (113 and 135 µg/L, respectively), indicating selenium replete populations. In the NPC study, stratification into tertiles, with lowest tertile <105.2 µg/L, which is still high, did not change the outcome of the study.

Later, Zhang et al. (76) conducted a meta-analysis of 16 prospective observational studies and nine randomised controlled trials (RCTs) up to the end of 2013. In the observational studies, a non-linear inverse association was found between risk of CVD and selenium status that was significant between 55 and 145 µg/L of circulating selenium at baseline. The meta-analysis of the RCTs failed show any effect of selenium supplementation. Again, it should be noted that among the RCTs with the highest impact were the American NPC and SELECT studies with high baseline selenium status.

Another systematic review and meta-analysis by Jenkins et al. (77) included 43 RCTs using antioxidants with and without supplementation of different chemical forms of selenium and lasting for 24 weeks or more. While no effects on cardiovascular mortality were seen in groups receiving antioxidants alone, the group receiving the combination with selenium showed a reduced cardiovascular mortality (relative risk [RR]: 0.77; 95% confidence interval [CI]: 0.62, 0.97; *P* = 0.02).

In a systematic review and meta-analysis, 13 studies with observational and RCT designs, CVD incidence and mortality were investigated (78). A decreased cardiovascular mortality and event incidence was observed in those with a high physiological status versus those with a low physiological status. There was a linear dose-response relationship between blood selenium concentration and CVD incidence. The risk was reduced by 15% per 10 µg increment in blood concentration. The highest blood selenium concentration in the study was 150 µg/L.

As noted above, a general problem of some meta-analyses of RCT was that no attention was paid to baseline selenium status in the analysis. Large American studies had a baseline selenium status that was generally much higher (>100 μg/L) than that of European studies. In a RCT in elderly Swedes with a mean baseline serum selenium value of 67 µg/L, intervention with selenium for 4 years reduced cardiovascular mortality for up to 10–12 years (79). Of interest, the impact of the intervention was significantly higher in the two lowest tertiles of baseline serum selenium (<65, 65–85, and >85 μg/L) (50).

In summary, observational and intervention studies conducted in populations low in selenium appear to show a cardiovascular risk reduction by increasing selenium intake, while large supplementation studies in selenium replete populations do not.

#### Type 2 diabetes

Early results in mice have shown a beneficial effect of selenium in diabetes (80, 81). The relationship between selenium and type 2 diabetes was investigated in two US RCTs with cancer as the primary endpoint, NPC and SELECT. In the NPC trial, an increased risk of type 2 diabetes was seen for the group supplemented with 200 μg/day Se of selenized yeast. Moreover, an increased risk of type 2 diabetes was observed for those in the upper tertile of baseline serum selenium (>122 μg/L) in comparison with those in the two lower tertiles (82). Notably, the increased risk of diabetes was observed in men only and was based on few cases. In the SELECT study, there was no increased risk of the supplemented group (200 μg/day as selenomethionine) (69). In addition, another RCT failed to show an association between selenium supplementation and an increased risk of type 2 diabetes, except among participants >63 years of age (RR = 2.21; 95% CI = 1.04 to 4.67, *P* = 0.03) (83). In a systematic review, Kohler et al. included 13 observational studies, three longitudinal, five case-control and five cross-sectional studies (84). Half of the longitudinal studies showed a positive association between selenium and type 2 diabetes, while most cross-sectional studies showed positive associations between plasma or serum selenium and type 2 diabetes or fasting blood glucose (85, 86). Vinceti et al. (87) conducted a systematic review and a dose-response meta-analysis on risk of type 2 diabetes and biomarkers of selenium exposure based on 34 observational studies (seven case-control, nine cohort, 18 cross-sectional). The association between risk of type 2 diabetes and dietary selenium was nonlinear, with risk increasing above 80 μg/day. Compared to whole blood/plasma/serum selenium concentrations of 90 μg/L, a value of 160 μg/L gave a risk ratio of 1.96 for type 2 diabetes.

Several mechanisms behind the apparent relation between selenium and type 2 diabetes have been suggested, such as overexpression of GPX1 with reduced H_2_O_2_ and compromised insulin signalling resulting in hyperinsulinemia and glycaemia (88). The risk of reverse causation especially in the cross-sectional studies should also be noted. Experimental evidence indicate that increased hepatic SELENOP synthesis, secretion and consequently increased plasma selenium could be a result of abnormal glucose metabolism rather than its cause (88, 89).

Beneficial effects and safety of selenium supplementation for type 2 diabetes were addressed in a systematic review of four RCTs, one from France and three from Iran (90). The authors concluded that the studies were heterogeneous with respect to i.a. duration and selenium compound used, and provided no consistent evidence of a beneficial effect of supplementation.

In summary, most cross-sectional studies showed significant positive associations between serum/plasma selenium and type 2 diabetes or fasting plasma glucose, but fewer longitudinal studies were consistent. Less evidence for a causal relationship between selenium supplementation and risk of type 2 diabetes was provided by dietary intervention studies. Hence, these studies cannot be used for setting DRVs.

#### Cancer

Many observational epidemiological studies with different designs have been conducted to investigate the preventive effect of selenium both on over-all and site-specific cancer risk. They have been summarised in systematic reviews and meta-analyses (91–93).

In addition, several intervention studies have been carried out. The NPC trial, in which older patients (*n* = 1,312) in the US with a history of basal and/or squamous cell carcinoma were given 200 μg Se/day as selenized yeast or placebo for preventing recurrence of cancer (94), demonstrated an increased risk of nonmelanoma skin cancer following selenium supplementation (95). The study authors suggested that exposure to arsenic containing pesticides could be a confounder. Possible effects on other cancer types were also investigated, and reduced incidences were found of lung cancer, colorectal cancer, and for prostate cancer particularly in men with low baseline selenium (94). Prostate cancer risk was further investigated in the SELECT that included 35,533 American men. An intervention was used 200 μg Se/day (as selenomethionine), alone or in combination with vitamin E (96). Selenium had no effect on over all prostate cancer risk (69), and among those with high selenium status, an increased risk of high-grade prostate cancer was found (97).

Observation studies and randomised controlled studies have been summarised in three recent systematic reviews and meta-analyses presented below.

The associations between serum or toenail selenium concentration and cancer risk were investigated using meta-analysis, meta-regression, and dose-response analysis. A total of 69 studies, both observational of variable design (*n* = 64) and clinical trials (*n* = 5) were included (91). While selenium supplementation did not reduce overall cancer risk in randomised controlled studies, high selenium exposure appeared be protective in observational studies. As regards specific cancer risks, high selenium exposure was associated with a decreased risk of breast cancer, lung cancer, oesophageal cancer, gastric cancer, and prostate cancer, but not with colorectal cancer, bladder cancer, and skin cancer.

Vinceti et al. (98), in an updated Cochrane review, evaluated the protective effect of selenium intake and cancer risk. They included 10 RCTs and 70 observational studies. No reduction in risk of overall cancer or specific cancers, mostly prostate cancer, was observed. In observational studies, the evidence of an association between selenium and cancer risk was inconsistent. Overall pooled data revealed an inverse relation between selenium exposure and risk of overall cancer or some specific cancers, such as colon and prostate cancer.

In a systematic review and meta-analysis of population-based prospective studies, the association between dietary intake of selenium and risk of different cancers were assessed (92). A total of 39 studies were included in the final analysis. Selenium intake was inversely associated with overall cancer risk. Selenium intakes of 55 μg/day and above decreased the risk of cancer. Extra selenium intake from supplements was protective at intake levels ≥55 μg/day.

The role of selenium in cancer prevention is still not clear despite numerous human studies. While RCTs provided little evidence for a protective effect, observational studies might suggest an inverse association between selenium intake or status and over-all cancer risk, but varying with specific cancers. Notably, the SELECT study, which had the highest impact in meta-analyses of the RTCs, had a high baseline selenium in plasma (136 µg/L) indicating that the population was replete prior to the intervention.

In summary, selenium seems to be protective against some cancers in populations low in selenium. The available studies cannot be used for setting of DRVs.

#### Total (all-cause) mortality

All-cause mortality has been investigated in observational follow-up studies, and it appears that there is an inverse association between mortality and serum selenium concentration and a reduced mortality at a selenium concentration of about >100 µg/L in studies both from the US and Europe (30, 99–101). In a study from China based on the Linxian study cohort, no association of baseline selenium with total deaths was observed, but 90% had a baseline serum selenium concentration below 94 µg/L (102). In the Linxian General Population Nutrition Intervention Trial, supplementation with selenium for 5.25 years was associated with a reduced all-cause mortality at the end of the study and 10 years, but not 25 years post-trial (103, 104). In a systematic review and meta-analysis, the group receiving selenium in combination with antioxidants showed a reduced all-cause mortality (RR: 0.90; 95% CI: 0.82, 0.98; *P* = 0.02), and not the group receiving antioxidants alone (77).

The studies on mortality provide evidence for a reduced all-cause mortality risk at a plasma selenium concentration of around 100 µg/L and above, but these data cannot be used for deriving DRVs.

### Other health related outcomes

The relationship between selenium status or intake and other health outcomes has also been investigated in observational studies and a few RCTs. The outcomes refer to immune functions and infectious diseases (105), fertility, reproduction and neurodevelopment (106–108), thyroid function and cognition. Although the data provide evidence for a positive relationship between selenium status and positive outcomes, the overall evidence is limited. In two recent systematic reviews and meta-analyses it was found that serum levels of selenium were lower in patients with gestational diabetes (109), and preeclampsia was associated with lower levels of selenium (110). The latter could not be confirmed in a Norwegian birth cohort (111). In the same cohort it was also found that maternal dietary selenium intake, but not intake of supplements or whole blood selenium concentration, was significantly associated with prolonged gestational length and decreased risk of preterm delivery (112). Furthermore, increased risk of neonatal morbidity or mortality was observed at a maternal dietary intake below 30 µg/day (107).

The evidence provided for other outcomes is not suitable for deriving DRVs.

## Requirement and recommended intakes

The main physiological role of selenium is to support the synthesis and maintenance of selenoproteins in tissues. The amount of selenoproteins is likely to be related to the total amount of proteins in the body. The daily requirement of selenium is assumed to depend on body size (40).

Observational studies provide evidence suggesting an inverse relationship between selenium status and risk of CVDs, cancer, and all-cause mortality at low levels of intake (<55 µg/day) and at low plasma or serum concentrations (<100 µg/L). Most intervention trials, which show no beneficial effect on health outcomes of selenium supplementation, have been carried out in ‘selenium-replete’-populations (>100 µg/L). From a nutritional point of view, this would rather not be expected. Some intervention studies that have been conducted in populations low in selenium show beneficial effect health outcomes. However, there is a lack of dose-response data. Hence, the observational and experimental studies in humans on health outcomes are not suitable for deriving dietary reference values.

Previous criteria for deriving DRVs have been ‘maximisation’ of GPX3 in plasma or GPX1 in blood platelets. More recently, realising that SELENOP plateaus at a higher plasma value than GPX (>110 µg/L vs. 70–90 µg/L), ‘optimising’ SELENOP in plasma was used by NNR2012 (40) and EFSA in 2014 (30). SELENOP in plasma is considered the most informative biomarker of selenium status based on its role in regulation and transport of selenium in the body and its response to selenium intake. It is assumed that maximum expression of SELENOP in plasma indicates adequate supply of selenium to all tissues and selenoproteins, and represents a saturation of the ‘regulated body pool of selenium’.

Whether a maximised expression of SELENOP or assumed saturation of the regulated pool of selenium is required to achieve an optimised health benefit is not known. Notably, however, the plasma concentration below which selenium is inversely correlated with risk of disease or mortality, 100 µg/L is close to the concentration at which SELENOP expression is maximised, 110 µg/L.

As discussed above, several studies have investigated the relationship between selenium concentration and SELENOP concentrations in plasma. The plasma concentrations of selenium required to achieve maximum SELENOP concentration varied between >90 and <130 µg/L (17). This is compatible with early studies on selenate and selenite supplementation showing a levelling off of selenium in plasma at a concentration of about 110 µg/L (20, 23), likely also representing SELENOP levelling off in plasma as inorganic selenium only supports selenoprotein synthesis and do not enter into other plasma proteins.

The selenium intake needed to achieve a plasma concentration of about 110 µg/L is likely to be dependent on selenium compound given. In the 40-week supplementation study in selenium deplete Chinese subjects weighing 58 kg, selenomethionine from supplement was their main source on top of an average intake of 14 µg/day (24). With total intakes of 49, 69 and 93 µg/day (0.85, 1.2 and 1.6 µg/kg and day), average plasma values ±SD of 98 ± 15, 112 ± 14, and 128 ± 15 µg/L, respectively was reached at 40 weeks. Mean SELENOP concentrations were 4.9, and 5.1–5.5 mg/L, respectively.

The results obtained in the Chinese study accord with results from a study of subjects in the UK. The participants had an estimated baseline dietary selenium intake of 55 µg/day and a mean plasma concentration of 95.7 ± 11.5 µg/L. Upon supplementation with 50 µg/day of selenium as selenized yeast (total selenium intake 105 µg/day), SELENOP concentration plateaued at 6 weeks with plasma value at 10 weeks of 118.3 ± 13.1 µg/L (29).

It is concluded that based on the Chinese study, an average daily intake of dietary selenium of about 1.2 µg/kg bw would be sufficient to achieve an adequate selenium concentration in plasma and maximised expression of SELENOP in plasma. Using BMI-adjusted body weight, the mean body weights for men and women, 75 and 65 kg respectively, the daily requirement of would be 90 µg for men and 80 µg for women.

It is noted that in Finland, an estimated average selenium intake of 88 µg/day in men and 68 µg/day in women led to an average selenium concentration in plasma of 110 µg/L (see section on Dietary intake).

### Requirements in infants, children, and adolescents

Information on selenium requirements for infants, children and adolescents is scarce, and no information to support specific values exists.

Regarding infants, the requirement could be derived by extrapolation from the content of breast milk from replete women.

For children’s and adolescents’ requirements it is assumed that they would be like that of adults in addition to selenium needed for growth and selenoprotein synthesis. The total requirements for children and adolescents could be derived by extrapolation from the value used for adults using the method described in EFSA 2014, namely extrapolated on a body weight basis with addition of growth factor based on protein requirements as the requirement for selenium is linked to selenoprotein synthesis (4, 113).

### Requirements in pregnancy and during lactation

Information to support requirements in pregnant and lactating women is limited.

#### Pregnancy

It is known that maternal concentrations of selenium in whole blood and plasma as well as GPX activity decrease during pregnancy (4). This fall in plasma levels is assumed at least to some extent to be due to an increase in plasma volume. In addition, transfer of selenium will take place to support selenoprotein synthesis in the foetus and placenta. Adaptive changes during pregnancy have been assumed, as Swanson et al. 1983 in a balance study in American women found that pregnant women tended to reduce urinary excretion, retaining on average 10 and 23 µg/day more than non-pregnant women in early and late pregnancy. Based on the content of selenium in lean tissue and an average amount of 5 kg is deposited during pregnancy, Swanson and co-workers (114) estimated that an average retention of selenium would represent about 3.5 to 5.0 µg/day during 280 days of pregnancy. In contrast, urinary excretion in New Zealand women low in selenium remained constant at a low level during pregnancy (115).

#### Lactation

Dietary selenium intake correlates with breastmilk selenium concentration (116) and during continued lactation, the selenium concentration in the mother’s milk is reduced over time (117) when selenium intake is less than 45–60 µg/day but remains unchanged at intakes of 80–100 µg/day (40). Based on an average selenium content of breast milk of 15 µg/L (varying from 11 to 38 µg/L (117)) and assuming a volume of 0.8 L/day, EFSA estimated daily loss during first 6 month of lactation to about 12 µg/day (4). Thomson et al. (115) in their study in New Zealand women low in selenium found a clear reduction in renal plasma clearance of selenium during the period postpartum up to 12 months. This was also observed in women that received selenium supplement.

## Conflict of interest and funding

No specific conflicts of interest are declared.

This work was funded by the Nordic Council of Ministers, Norwegian Directorate of Health and Norwegian Institute of Public Health.
